# Potential Spatial Distribution of the Newly Introduced Long-horned Tick, *Haemaphysalis longicornis* in North America

**DOI:** 10.1038/s41598-018-37205-2

**Published:** 2019-01-24

**Authors:** R. K. Raghavan, S. C. Barker, M. E. Cobos, D. Barker, E. J. M. Teo, D. H. Foley, R. Nakao, K. Lawrence, A. C. G. Heath, A. T. Peterson

**Affiliations:** 1 Department of Diagnostic Medicine & Pathobiology, College of Veterinary Medicine, Kansas State University, Manhattan, 66506 Kansas USA; 2 Department of Parasitology, School of Chemistry and Molecular Biosciences, The University of Queensland, St. Lucia, QLD 4072 Australia; 3 Department of Ecology and Evolutionary Biology and Biodiversity Institute, College of Liberal Arts and Sciences, University of Kansas, Lawrence, 66045 Kansas USA; 4 School of Veterinary Science, The University of Queensland, Gatton, QLD 4343 Australia; 5 Division of Entomology, Walter Reed Army Institute of Research, 503 Robert Grant Avenue, Silver Spring, Maryland 20910 USA; 6 Department of Disease Control, Graduate School of Veterinary Medicine, Hokkaido University, Sapporo, 060-0818 Hokkaido Japan; 7 School of Veterinary Science, Massey University, Palmerston North, 4442 New Zealand; 8 Agresearch Ltd., c/o Hopkirk Research Institute, Private Bag 11008, Palmerston North, 4442 New Zealand

## Abstract

The North American distributional potential of the recently invaded tick, *Haemaphysalis longicornis*, was estimated using occurrence data from its geographic range in other parts of the world and relevant climatic data sets. Several hundred candidate models were built using a correlative maximum entropy approach, and best-fitting models were selected based on statistical significance, predictive ability, and complexity. The median of the best-fitting models indicates a broad potential distribution for this species, but restricted to three sectors—the southeastern United States, the Pacific Northwest, and central and southern Mexico.

## Introduction

The east Asian tick, also known as the long-horned tick, bush tick (Australia), and cattle tick (New Zealand), *Haemaphysalis longicornis* Neumann (Acari: Ixodidae) is native to Japan, China, Primorsky Krai region of eastern Russia, and Korea; and is well-established as an invasive species in Australia, New Zealand, and on several Pacific Islands^1,2^. The long-horned tick has been intercepted at US ports-of-entry several times over the years, but it was first confirmed to be present outside quarantine on a sheep in a New Jersey farm in November 2017 (State of New Jersey, Department of Agriculture, 2018). In quick succession, additional ticks were identified in May 2018, on a beef-cattle farm in Virginia; in the same month, ticks collected on two West Virginia farms (Hardy County, along the Virginia border) were also confirmed as *H. longicornis*. All immature life stages of the tick were found on the New Jersey farm, confirming that these ticks are able to survive local conditions and establish viable populations.

Serious concern exists that *H. longicornis* will successfully establish invasive populations in the US, and spread broadly from its current focus. In Australia, *H. longicornis* continues to spread to new regions: in about 1983 it spread from northwestern New South Wales and/or southeastern Queensland across Australia to the coastal areas near Perth in Western Australia^2^. Such invasion in the US would carry significant economic burden and human suffering, given that this tick is able to transmit various pathogens, including *Theileria orientalis* var. Ikeda and severe fever thrombocytopenia syndrome (SFTS) virus but potentially also many of the currently circulating tick-borne pathogens in North America. Indeed, these ticks may already have invaded more areas than has been appreciated so far, and indeed even broader swaths of North America may be accessible to this species if it is able to disperse *via* movements of birds. The livestock and dairy cattle trade in the US is highly networked, and high levels of interstate movement of animals occur each day. Transportation of *H. longicornis via* movement of livestock and other domestic animals is not currently monitored or regulated, so it is only a matter of time, in our opinion, that this species will spread further in North America.

In this study, we used the broad Eastern Hemisphere distribution of this species as an arena in which to calibrate ecological niche models for *H. longicornis*, and used those models to estimate the distributional potential of the species across North America. These models are based on the species’ distributional responses to relatively coarse-grained, abiotic, non-interactive environmental variables^3^, and as such do not take into account the distributional effects of biotic interactions. Still, ecological niche models have proven to have good predictive abilities as regards species invasions^4,5^, and a general idea of the tick’s invasive potential can inform efforts towards mitigation and eradication.

## Results

**Figure 1:**
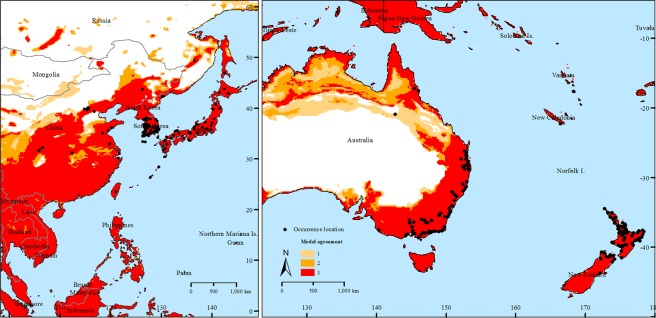
Known occurrence locations (black dots) of *Haemaphysalis longicornis*, used for niche modeling and partial geographic suitability in its native range.

Occurrence records from our surveys included 442 locations in Australia, New Zealand, China, Japan, Korea, Russia, and Taiwan. Twenty of these records did not include accurate location information and were therefore removed. A further 50 records, representing duplicates at single locations, were also removed, leaving 372 records. The Walter Reed Biosystematics Unit (WRBU) dataset contained 10,423 occurrence records, including two recent updates with 7 records. However, no uncertainty information was available for these updates and for 23 additional records, and two among the remaining records had uncertainty >10 km; all of these records were excluded. Most dramatically, 10,094 records represented specimen level duplicate records at single locations, leaving 304 unique data records for analysis. Our steps to avoid spatial autocorrelation in the occurrence dataset resulted in the removal of different numbers of occurrence points with different distance filters: 11 of the 304 occurrences were randomly removed with the application of 20 km filter distance, 81 occurrences with 35 km filtering, and 52 occurrences with 50 km filtering. This step resulted in 204 unique occurrence points that were used for model calibration and evaluation (Fig. 1).

The correlation matrix of variables revealed high correlations (*r* ≥ 0.8) among several variables (see Appendix 1). Based on these correlations, and guided by the variable importance information, bioclimatic variables 6, 16, 1, 5, 4, 7, 11, and 14 were removed in sequential passes. Subsequently, in two more jackknife models, bioclimatic variables 3 and 13 were removed. Variable reduction steps are detailed in Table 1.

**Table 1:** Summary of variable selection process using iterative jackknife analysis procedure in Maxent.

Jackknife step/variable set for modeling	Variables included	Variables removed
Step 1	All except bioclim 8, 9, 18, 19	1, 4, 5, 6, 7, 11, 14, 16,
Step 2/variable set 1	2, 3, 10, 12, 13, 15, 17	3
Step 3/variable set 2	2, 10, 12, 13, 15, 17	13
Step 4/variable set 3	2, 3, 10, 12, 15, 17	

**Figure 2:**
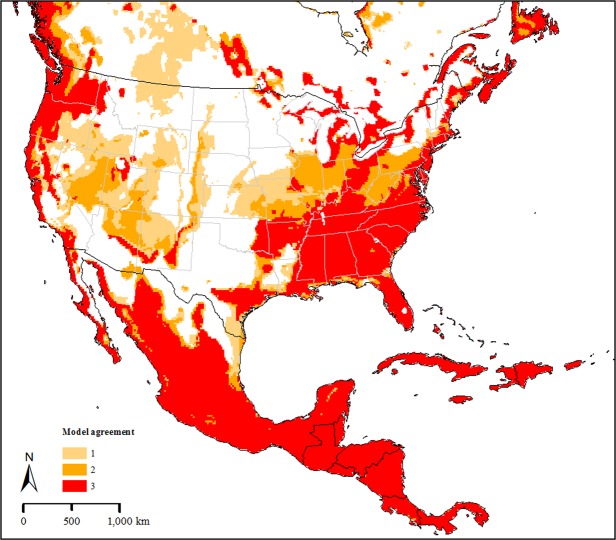
Predicted suitable areas for *Haemaphysalis longicornis* across North America. 1, 2, and 3 represent areas that were predicted to be suitable for the establishment of *H. longicornis* in North America by one, two and three models, respectively. Darker areas represent progressively higher agreement between the models.

**Figure 3:**
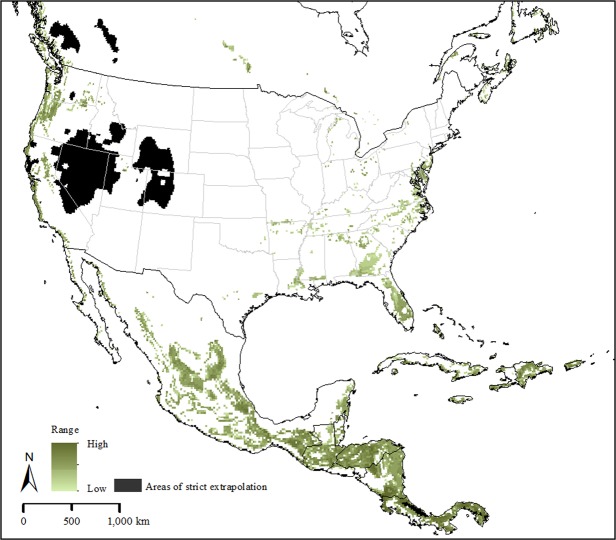
Uncertainty associated with the predicted suitable areas of *Haemaphysalis longicornis* across North America. Area in black represent uncertainty due to model extrapolation based on mobility-oriented pathway analysis; and, areas in light to darker shades of green indicate progressively higher uncertainty based on the range (maximum – minimum) of predicted probability for *H. longicornis* in the study region.

**Figure 4:**
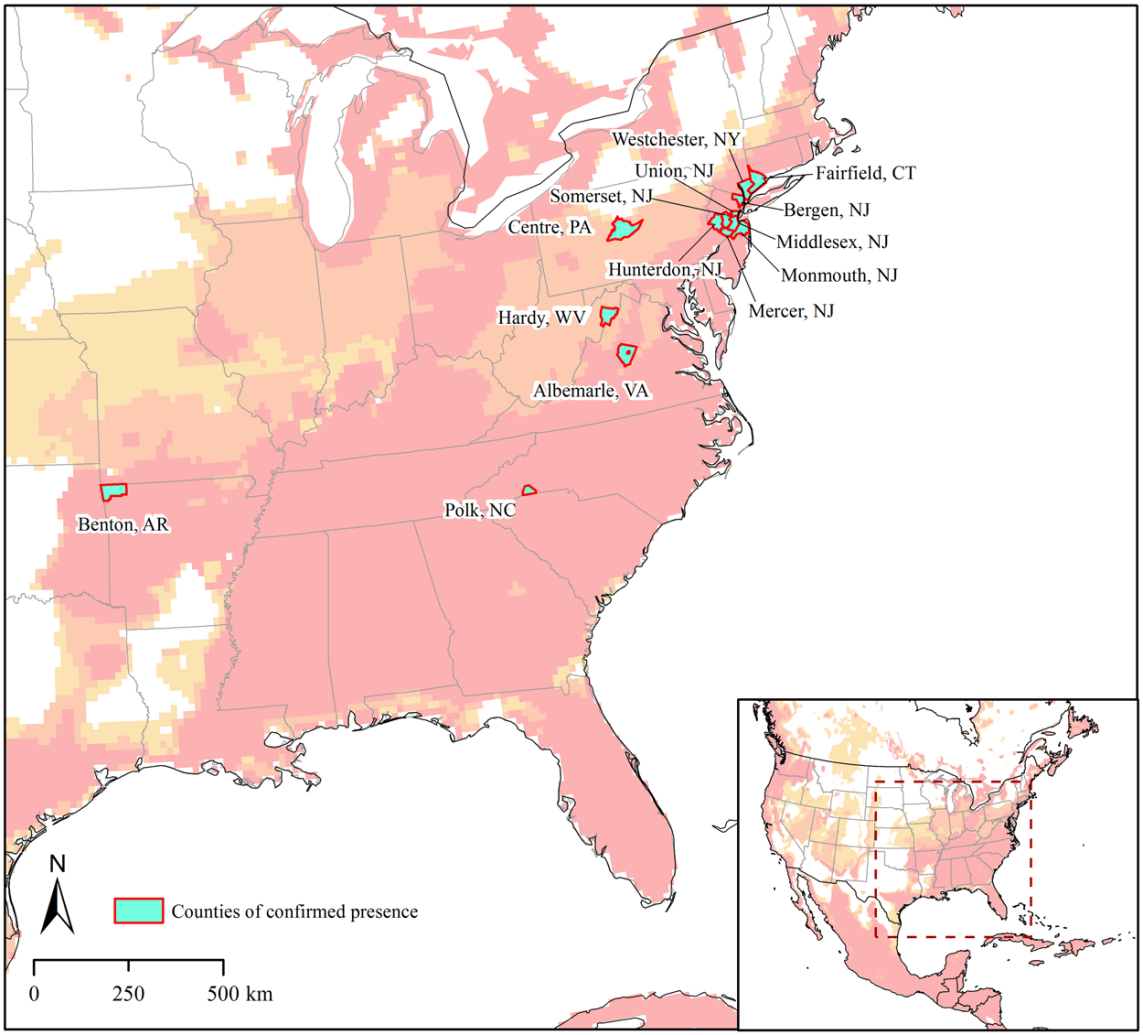
Counties in the United States in which the *Haemaphysalis longicornis* has been positively confirmed as of September, 2018. Colours in the background show areas that are predicted suitable for the establishment of *H. longicornis* in North America; darker shades indicate higher degree of agreement among models.

Our model selection exercises for each of the three occurrence data sets arrived at the same set of parameters; in each case a single candidate model was chosen. The median pixel values of the three models revealed a potential native distribution that rimmed coastal areas from Australia and New Zealand north to eastern Russia, with a broader interior distribution in China and Southeast Asia (Fig. 1). Transferring the models to North America identified three main areas: the southeastern US, the West Coast and more broadly in the northwestern US; and tropical areas of Mexico and Central America (Fig. 2). Uncertainty in these model predictions was focused in peninsular Florida, the northwestern US, and most notably in Mexico (Fig. 3); areas of strict extrapolation were confined to the interior American West. Overlaying known North American occurrences indicated that all fall within the geographic predictions of our ecological niche models in the eastern US (Fig. 4).

## Discussion

Ecological niche models represent a correlative approach to estimating a highly complex and challenging object, the fundamental ecological niche^3^. Difficult elements in this process include the limited representation of the universe of possible environments, possible confounding and confusing effects of biotic interactions, and the multidimensional nature of ecological niches. Furthermore, these elements may or may not be different between native ranges and invaded ranges, which complicates model transfers further^6,7^. In the case of *H. longicornis*, our models were likely compromised by uneven availability of known occurrences across the calibration region of the species, particularly in China (Fig. 1). Although our models were clearly not overfit to the available occurrence data (see, e.g., Peterson *et al*. 2014)^8^, the reality of their broader predictions as regards the occupied geographic area of the species can be evaluated only with additional occurrence data from key regions. Transferring the models did not appear to be compromised overmuch by concerns about model extrapolation (Fig. 3); however, our three models did produce potential distributional estimates that differed somewhat (Fig. 2), so some uncertainty remains in our North American projections.

The North American projection for *H. longicornis* reveals a significant and immediate concern for public health and livestock farming in the US and its neighbouring countries. The infection status of these ticks when they were found in the US has so far not been made public, and may not have been analysed; however, *H. longicornis* has been implicated, in at least some of its broad range, in transmission of *Theileria orientalis* var. Ikeda and *T. orientalis* var. Chitose, blood parasites that causes theileriosis to cattle^9–11^, an economically significant disease in Australia and New Zealand that has clinical symptoms that are similar to bovine anaplasmosis. Recently, *H. longicornis* was also implicated in transmission of SFTS virus to humans in South Korea and China^12–14^. The epidemiological role of long-horned tick in tick-borne encephalitis (TBE), a widely prevalent disease in Europe, the Russian Federation, and parts of East Asia is not entirely clear, but TBE virus has been isolated recently from *H. longicornis* and one of its mammalian hosts, striped field mouse, *Apodemus agrarius* in South Korea^15–17^. The presence of DNA of *Ehrlichia chaffeensis*, *Anaplasma phagocytophilum*, and *A. bovis* was recorded in a recent study of field-collected *H. longicornis* in South Korea^18^. Successful transovarial transmission of *Babesia ovis* in this tick species was demonstrated by Ohta *et al*.^19^ under laboratory conditions, and successful transovarial and transstadial transmission of *B. equi* occurred in a different study^20^, indicating that *H. longicornis* may play a role in the transmission of these pathogens to cattle and horses in Japan.

In China, where Lyme disease is widely prevalent, *H. longicornis* appears to play a role in the epidemiology of this disease. In a study that evaluated 17,000 ticks collected between 1987 to 1997, *Borrelia burgdorferi sensu lato* was found among several tick species using direct immunofluorescence assay^21^. In that study, *H. bispinosa* and *Ixodes granulatus* were determined to be the primary tick vectors of *B. burgdorferi sensu lato*; recently, however, it was determined that all of the *H. bispinosa* ticks reported in China are in fact *H. longicornis*^22^. Subsequent studies have found co-infected adult *H. longicornis* with Lyme spirochetes and spotted fever group (SFG) rickettsiae^23^, and several ticks collected in eastern China were PCR-positive for *B. burgdorferi sensu lato*^24^.

Some of the biological traits of *H. longicornis* are particularly concerning in the context of its establishment potential and likely further expansion from its current distribution in North America. First, *H. longicornis* can reproduce both sexually and asexually; in fact, obligate parthenogenesis is likely the only reproductive mechanism of this species in Australia and New Zealand, as male ticks of this species in these countries have been found rarely or not at all^11^. Although parthenogenetic reproduction has disadvantages, such as low genetic variance in the population and associated consequences, this trait could be advantageous as females in the initial population do not require mates, and yet produce large numbers of offspring. Examples of this can be observed among some invading aphids (e.g., *Myzus persicae*)^25^, sawfly (*Nematus oligospilus*)^26^, and hemlock wooly adelgid (*Adelges tsugae*)^27^.

Second, in its native range, *H. longicornis* is a host generalist, associated with a broad range of host species^11,28^, including many livestock animals that are also present in the U.S that may readily serve as hosts and facilitate the tick’s establishment. All known occurrences of *H. longicornis* in the US so far have in fact been on livestock animals. Detecting wildlife parasitization by this species once it is established will be difficult, and will require considerably more resources as potentially many hosts exist. Different wildlife hosts in New Zealand include the brown hare (*Lepus europaeus*), four species of deer (fallow, *Dama dama*; red, *Cervus elaphus scoticus*; rusa, *C. timorensis*; and sambar, *C. unicolor unicolor*), and among birds, the North Island brown kiwi (*Apteryx mantelli*) and buff-banded rail (*Gallirallus philippensis*)^11,29^. In South Korea, the raccoon dog (*Nyctereutes procyonoides*), Chinese water deer (*Hydropotes inermis*), Siberian roe deer (*Capreolus pygargus*), and Asian badger (*Meles lucurus*) are terrestrial mammalian hosts^30^, and ruddy kingfisher (*Halcyon coromanda*), fairy pitta (*Pitta nympha*), white’s thrush (*Zoothera aurea*), and grey-backed thrush (*Turdus hortulorum*) are among the migratory birds known to carry *H. longicornis* (Choi *et al*.)^31^. This list is not meant to be an exhaustive inventory of wildlife-hosts associated with *H. longicornis*; most definitely, it does not include every species with which *H. longicornis* may be associated across its native range, but it is indicative that this tick are likely to adapt to many types of wildlife hosts in North America, given its broad host adaptive capacity in its native range.

Finally, the current geographic distribution of *H. longicornis* shows that it has been able to adapt successfully to a wide range of climate types, ranging from the equatorial Papua New Guinea and Pacific Islands to the conditions of Primorsky Krai region in Russia, with a much more seasonal climate^11^. These ticks have also been found at relatively high elevations, up to 526 m in Australia^32^ and at 952 m in China^33^. Like many North American ixodid ticks, *H. longicornis* can diapause when the conditions are not favourable, and use this ability as an effective mechanism to withstand adverse conditions^11,32^. Vast areas of North America, particularly the southeastern US, the West Coast and broad areas of the northwestern US, are identified by our model as climatically suitable for establishment of this species, with the only limitation to establishment being access to the disparate potential distributional areas.

This species disperses naturally with the assistance of infested mammals and migratory birds, but it can also colonize new areas rather quickly with the help of humans. In the worst case, infested pets such as dogs could be transported across the country within a matter of hours *via* airplanes. Dogs are of particular concern for several reasons. First, dogs are suitable hosts for *H. longicornis*^1,2^. Second, the pet dogs of a farm-worker were the most likely carriers of *H. longicornis* from New South Wales/southeast Queensland to Walpole in Western Australia when this tick invaded Western Australia in about 1983 (Besier personal communication to SC Barker). Third, there is precedence for *H. longicornis* on dogs reaching the US, albeit not mainland US, but Hawaii: a living female *H. longicornis* was shipped to Hawaii from Australia on a sheep dog destined for Texas in 1967^1^. What should not be discounted also is that humans can be important carriers of ticks between countries^34^. Transportation of livestock, particularly beef and dairy cattle in cattle trains by road and rail, is another highly likely scenario by which this tick species can disperse broadly with human assistance. Cattle trains move slowly and stop frequently *en route* to their destinations, potentially dropping female ticks along the way. Strict inspection and quarantine of cattle, other livestock, and pet animals, particularly dogs, about to be transported from the current focus areas of this tick are therefore necessary. The health burden to humans and animals associated with ticks in general is increasing steadily in the US^35,36^, and various tick species are expanding their distributions to the north and west^37–39^. The invasion of *H. longicornis*, with its broad capacity to establish and transmit diverse pathogens, represents a significant addition to this existing burden.

## Methods

### Occurrence data

Occurrence data for *H. longicornis* used in this study were drawn from two sources: data housed in the Walter Reed Biosystematics Unit (WRBU) and our own records (field collections in Australia by Stephen and Dayana Barker, and Ernest Teo, Japan by Ryo Nakao; in South Korea by WRBU and US military personnel, and New Zealand by Allen Heath). The date range of the occurrence data spanned 1990–2017. Geographic coordinates for these occurrences were available in both datasets in decimal degrees; WRBU data also included a measure of uncertainty (in meters) for most records. Data from both sources were checked carefully for duplicate occurrences, and for any potential errors in locational information. Areas for calibrating the niche models that represent areas accessible to the species through dispersal, termed **M**^40^, were estimated as areas within 5° (≈550 km) of known occurrences using ArcGIS 10.5.1; these areas were modified posteriorly to remove the Philippines and areas east of Wallace’s Line in Indonesia, as they were located across strong dispersal barriers for the species and are not known to hold populations. This represents areas that would be accessible to the species for establishment based on their natural dispersal ability. We considered several distances, but collectively agreed that 5° is a close approximation since this species is not sampled thoroughly in many parts of its native range.

Survey-based occurrence data are often clustered in geographic space, and models built with such data are prone to produce erroneous results due to spatial autocorrelation. To reduce spatial autocorrelation among occurrence points, we filtered occurrences as follows. The environmental heterogeneity within the **M** area was evaluated using the Spatial Distribution Modeling SDMToolbox 1.1c in ArcGIS^41^. The construction of this heterogeneity layer is done by analysing how environmentally variable is each area in the **M** using a moving window approach and the 3 first principal components of the environmental variables. Environmental heterogeneity was reclassified into three broad classes using Jenk’s Natural Breaks classification method^42^, representing low (class 1), medium (class 2), and high (class 3) heterogeneity. Occurrence points were then filtered using different proximity values in each of the three classes; for occurrences in class 1, points <50 km from each other were removed; for points within class 2, 35 km was used as a filter distance; and for class 3, 20 km was used in the distance filter. This procedure was repeated three times to obtain three different sets of occurrences, given random choices of which point should be the representative point, using the spThin library in R Statistical Package 3.5.0^43,44^.

We used these filter distances primarily considering three factors: (1) the pixel resolution of the environmental variables (~17 km), (2) the uncertainty of georeferenced records, and (3) the environmental heterogeneity in each class. Using the shortest distance (20 km) diminished the effect of spatial autocorrelation problems in zones that are highly heterogeneous, but since this distance is not too long, it also allowed us to appropriately represent the environmental space occupied by the species in these areas. The second distance (35 km) diminishes problems of spatial autocorrelation and it was used in areas with medium levels of environmental heterogeneity; therefore, there was no concern with regards to losing details that closer records will otherwise reconstruct. The third distance (50 km) was used in areas with low levels of heterogeneity, where problems of spatial autocorrelation can be more problematic, and hence a longer distance. In this last case, the environments used by the species will be appropriately reconstructed by widely separated records because these areas are more homogeneous.

### Environmental data

We used bioclimatic variables available from MERRAClim^45^ (https://datadryad.org//resource/doi:10.5061/dryad.s2v81) to summarize environmental landscapes across the species’ current geographic range and invaded landscapes. This climate dataset was built from hourly data of temperature (2 m above ground) and specific humidity, and has global coverage at different spatial resolutions. For this study, we used the 10′ (~17 km) resolution, which more or less matches the uncertainty associated with the occurrence data (10 m – 10 km). MERRAclim data are comparable to other bioclimatic datasets based on ground station interpolations, except that water availability is expressed in terms of humidity rather than rainfall^46^.

The relevance of bioclimatic layers to the distribution of *H. longicornis* in its current geographic range (Australia, China, Japan, Korea, New Zealand and eastern Russia) was screened using a combination of the jackknife procedure^47^ in Maxent and the low collinearity among candidate variables. First, we excluded variables 8, 9, 18, and 19 *a priori* since these variables hold odd spatial artifacts^48^. For the remaining 15 candidate variables, correlations among variables were estimated using R Statistical Package 3.5.0. We also calibrated a preliminary niche model, selecting the jackknife option. Based on the variable contribution estimates generated by the jackknife plot and the correlation coefficients, we evaluated which variables to retain for further evaluations. If two variables were correlated (*r* ≥ 0.8), a highly contributing variable was preferred over the other. We proceeded to fit several models in sequential steps using the jackknife procedure in Maxent, each time removing variable(s) that were weak contributors to the overall quality (gain) of the respective model, or that did not make significant independent contributions to the model. Three sets of bioclimatic variables were retained after these jackknife screening steps and were used for constructing niche models as explained below.

### Niche modeling

We used Maxent 3.4.1 for developing models^47^. In view of the significant sensitivity of this algorithm to particular parameter settings^49,50^, we conducted a detailed model selection exercise, using the *kuenm* R package (Cobos *et al*., submitted). Briefly, we explored all combinations of (a) 17 values of the regularization parameter (0.1–1.0 at intervals of 0.1, 2–6 at intervals of 1, and 8 and 10), (b) 29 sets of response types in the models (all potential combinations of five feature classes: linear, quadratic, product, threshold, and hinge), and (c) 3 sets of environmental variables (see above). As such, a total of 1,479 models were calibrated for each occurrence data set, and each model was evaluated for statistical significance (partial ROC tests)^51^, performance (omission rate)^52^, and the Akaike Information Criterion corrected for small sample sizes (AICc)^49^. Final models were selected under the three criteria (first, statistical significance, then omission rate, followed by AICc) (Cobos *et al*. submitted).

We used the best parameter settings chosen for the three occurrence data sets to create final models. These final models, though only calibrated across **M**, were transferred globally, and particularly to North America, which is the focus of this contribution. To summarize predictions across the three final models, we calculated median values for each pixel, and assessed uncertainty in the model outputs as the range of the medians of 10 replicate analyses for each model. To provide a further check on the reliability of our model transfers, we calculated the mobility-oriented parity (MOP) metric^6^, to offer a view of the novelty of environmental values and combinations of values across the transfer area, with special attention paid to areas of strict extrapolation. Finally, we assembled the still relatively few known North American records and overlaid them on the final models, as a means of checking, at least qualitatively, the reality of the North American predictions.

## Data Availability

Data used in conducting this study are available for researchers upon request to the corresponding author for reasonable use in research.
